# Available Information

**Published:** 1914-07

**Authors:** 


### Abstracts and Selections.

### Available Information.

The American Social Hygiene Association, whose address is 105 West Fortieth Street, New York City, is the most potent factor in our country for the dissemination of information on the social questions. The following announcement, therefore, may be of value to those who desire further knowledge:

"Among the means by which the American Social Hygiene Association hopes to be of special service to all local societies promoting social hygiene work, the directors believe that the reference library maintained at the national office is not the least important. In addition to books received by purchase, or through the courtesy of publishers, authors, or donors, the library will maintain an index of available books, monographs, magazine articles, stories, etc., within the field of the Association's work. A series of traveling libraries on the moral, social, economic, medical and educational phases of the work is being planned for co-operation with the local libraries and other agencies throughout the United States.

"The librarian will be glad to correspond with persons requiring special references and data. The more important new books and articles on social hygiene will be available for reference by visitors and matter important for special purposes will be found briefly abstracted for more ready reading. The librarian will prepare book reviews, which will be sent to members and to others placed on the mailing lists.

"The library begins its work with 564 volumes and approximately 1500 pamphlets in English, German, French, and other languages. It is hoped that a special library fund may soon be obtained to insure the rapid development of this important part of the Association's work. A questionnaire has been sent to all libraries in the United States comprising more than five thousand volumes, requesting lists of books catalogued under social hygiene subjects and inviting correspondence.

"The library is open daily except Sundays and holidays from 9 a. m. to 5 p. m., and is at the service of those who are interested in social hygiene."

